# Biodegradation of Polyvinyl Chloride by *Acinetobacter* sp. Isolated from Soil

**DOI:** 10.3390/microorganisms14102332

**Published:** 2026-10-13

**Authors:** Shumao Chai, Jiahao Wang, Yiru Zhang, Zihan Zhang, Zihan Yang, Yitong Wei, Mingfu Niu

**Affiliations:** 1 College of Food and Drug, Luoyang Normal University, Luoyang 471934, China; 232364003@lynu.edu.cn (J.W.); 252382102@lynu.edu.cn (Y.Z.); 252354014@lynu.edu.cn (Z.Z.); 252354046@lynu.edu.cn (Z.Y.); 252354024@lynu.edu.cn (Y.W.); 2 College of Food and Bioengineering, Henan University of Science and Technology, Luoyang 471023, China; mingfuniu@163.com

**Keywords:** polyvinyl chloride, biodegradation, *Acinetobacter*, catalase-peroxidase

## Abstract

The excessive use of plastics has seriously damaged the environment. Biodegradation of plastics, especially polyvinyl chloride (PVC), by microorganisms has become a promising strategy for the remediation of environmental pollution. Plastic-contaminated soil is an important source for isolating microorganisms with plastic-degrading ability. In this study, a PVC-degrading strain, designated PVC-3-16, was isolated from farmland soil and identified as *Acinetobacter calcoaceticus*. After 60 days of incubation, a new characteristic functional group was detected on the surface of PVC powder by attenuated total reflection Fourier transform infrared spectroscopy. A putative catalase-peroxidase enzyme derived from the genome of PVC-3-16 was successfully expressed and purified. After treatment with the catalase-peroxidase, hydroxyl groups and cracks were detected on the surface of PVC films by attenuated total reflection Fourier transform infrared spectroscopy and scanning electron microscopy, respectively. The M_n_, M_w_, and M_z_ values of the PVC films decreased by 10.7%, 9.4%, and 5.7%, respectively, after treatment. The degradation product catalyzed by the catalase-peroxidase was identified as C_21_H_44_O. These findings indicate that PVC-3-16 is a promising strain that can be used for PVC biodegradation.

## 1. Introduction

Plastic products are widely used in food packaging, textile fibers, and construction materials due to their low cost, light weight, good stability, and chemical inertness. The main polymers in plastic products are polyethylene (PE), polyurethane (PU), polypropylene (PP), polyethylene terephthalate (PET), polyvinyl chloride (PVC), and polystyrene (PS) [1]. The increasing demand for plastic products and their large-scale production have significantly contributed to environmental pollution [2]. Currently, the main methods used for managing plastic waste are landfill, incineration, recycling, and biodegradation [3]. Owing to their low cost, landfill and incineration are widely used waste management methods in developing countries [4]. Previous studies have shown that the content of microplastics (particles smaller than 5 mm) in landfill areas is up to 7% of the soil dry weight [5]. Microplastic accumulation in landfills can directly affect the terrestrial and aquatic environments, ultimately posing risks to human health. Although incineration can completely degrade plastic waste, it releases harmful gases that pollute the atmosphere. Particularly, PVC can release chlorides and generate chlorinated dioxins during incineration [6]. Therefore, researchers are now focusing on the biodegradation of plastics to minimize environmental harm.

The biodegradation process of plastics is generally divided into four stages: (1) biodeterioration, in which microorganisms form biofilms on the plastic surface, altering the local pH and causing surface damage; (2) fragmentation, in which plastics are broken down by enzymes, such as peroxidases, laccases, and esterases secreted by microorganisms or released from disrupted microbial cells; (3) assimilation, in which oligomers and monomers are transformed and transported into microbial cells; and (4) mineralization, in which small molecules are utilized as carbon sources for cell growth [7]. Owing to their high molecular weight and strong hydrophobicity, plastics are recalcitrant to biodegradation. Nonetheless, researchers have isolated and characterized several microorganisms and enzymes capable of degrading plastics. Muhonja et al. isolated two PE-degrading strains, *Aspergillus oryzae* and *Bacillus cereus*, and reported that laccase, esterase, and alkane hydroxylase were the key enzymes involved in PE biodegradation [8]. Gao et al. isolated a PE-degrading marine fungus and verified PE degradation by laccase and peroxidase [9]. Similarly, a PE-degrading bacterium was isolated from Antarctic sea ice, and a laccase with verified PE-degrading activity was subsequently obtained from this strain [10]. Interestingly, the laccase retained 54.3% of its activity at 0 °C, indicating that it is a cold-active enzyme [10]. In addition to peroxidases and laccases, cutinase can also depolymerize PET [11].

Extensive research has been conducted on the biodegradation of PE and PET. However, studies on PVC biodegradation remain scarce. Previous studies have identified several microorganisms with PVC-degrading ability. Giacomucci et al. identified two PVC-degrading strains, *Pseudomonas citronellolis* and *Bacillus flexus*, and reported that PVC films incubated with *P. citronellolis* for 30 days showed a weight loss of 19% [12]. Similarly, PVC films incubated with the *Klebsiella* sp. strain EMBL-1, isolated from the larval intestine of *Spodoptera frugiperda*, showed a weight loss of up to 19% [13]. However, the PVC films used in these studies contained plastic additives. Thus, the above-mentioned weight loss of PVC films cannot be entirely attributed to cleavage of the PVC backbone. Consequently, studies using additive-free PVC for the isolation of microorganisms have rarely been reported. Khandare et al. isolated the marine bacterial strains T-1.3, BP-4.3, and S-237 belonging to the genera *Vibrio*, *Alteromonas*, and *Cobetia*, respectively, all of which exhibited the ability to degrade additive-free PVC. Strain BP-4.3 showed the highest degradation ability, with 1.7% weight loss of PVC films after 60 days of incubation [14].

Soil is an important reservoir of microbial diversity, and a number of microorganisms with PVC-degrading ability have been isolated from soil. Polluted soils, such as landfill and farmland soils, have attracted increasing attention from researchers. Metagenomic analysis of PVC-buried soil showed that the Proteobacteria and Actinobacteria were dominant phyla, suggesting that these phyla may play important roles in the biodegradation of PVC [15]. Several PVC-degrading bacterial strains, including *Bacillus licheniformis*, *Achromobacter xylosoxidans* [16], *Streptomyces gobitricini* [17], and *Bacillus toyonensis* [18], have been isolated from polluted soils. In addition to bacteria, the fungal species *Aspergillus niger* and *Aspergillus glaucus*, both of which exhibit PVC-degrading ability, have been identified [16].

Despite the identification of a number of microorganisms capable of degrading PVC, the PVC degradation mechanism remains unclear. Catalase-peroxidases have been reported to depolymerize the main chain of PVC [13], and laccases are involved in both PE and PVC degradation [19,20,21]. Zhang et al. reported two laccases with PVC-degrading ability [21]. The presence of chlorine in PVC limits its degradation compared with other non-chlorinated polymers. Hence, dechlorination catalyzed by enzymes is the critical step in the PVC degradation pathway [21].

In this study, a bacterial strain was isolated from farmland soil. The degradation ability of this strain was supported by attenuated total reflection Fourier transform infrared spectroscopy (ATR-FTIR). To identity the enzymes involved in the biodegradation of PVC, the genome of this strain was sequenced and analyzed, and a putative catalase-peroxidase was successfully expressed and purified. The PVC-degrading ability of purified catalase-peroxidase was assessed by ATR-FTIR, scanning electron microscopy (SEM), gel permeation chromatography (GPC), and gas chromatography-mass spectrometry (GC-MS). The results of this study provide new insights into the microbial degradation of PVC.

## 2. Materials and Methods

### 2.1. Soil Sample and Strains Isolation

A soil sample was acquired from a plastic-contaminated farmland in Weifang, Shandong Province, China (37°7′ N, 119°5′ E) at a depth of 30 cm. Five grams of soil were added to 45 mL of sterile water and incubated at 30 °C for 24 h with shaking at 150 rpm in a shaker (Changzhou Nuoji Instrument Co., Ltd., Changzhou, China). The soil suspension (1 mL) was then added to 100 mL of mineral salt medium (MSM) containing 1% (*w*/*v*) pure PVC powders (purchased from Shanghai Macklin Biochemical Technology Co., Ltd., Shanghai, China) as the sole carbon source. The composition of the MSM was as follows: (NH_4_)_2_SO_4_ 2.0 g/L, Na_2_HPO_4_·12H_2_O 1.5 g/L, KH_2_PO_4_ 1.5 g/L, MgSO_4_·7H_2_O 0.2 g/L, CaCl_2_·2H_2_O 0.01 g/L, FeSO_4_·7H_2_O 0.001 g/L, pH 7.0. Unless otherwise stated, all chemicals and reagents used in this study were purchased from Sangon Biotech (Shanghai) Co., Ltd., Shanghai, China. The soil-MSM mixture was incubated in a shaker (150 rpm) at 30 °C, and the enrichment culture was maintained for 90 days with subculturing into fresh MSM every 15 days. The enriched suspension was diluted and spread onto Luria Broth (LB) agar medium consisted of tryptone (10 g/L), yeast extract (5 g/L), NaCl (10 g/L), and agar (20 g/L). The inoculated LB agar plate was incubated at 30 °C for 2 days in a constant temperature incubator (Shanghai ZHICHENG Analytical Instrument Manufacturing Co., Ltd., Shanghai, China). After 2 days, the bacterial colonies were individually picked and inoculated into 100 mL of MSM, followed by incubation at 30 °C for 30 days. Strains capable of growing in MSM with PVC as the sole carbon source were selected for further study.

### 2.2. Assessment of PVC Biodegradation by Isolated Strains

To evaluate the degradation ability of the isolated strains, PVC powders were soaked in absolute ethanol for 36 h and then washed three times with 75% (*v*/*v*) ethanol. The strains were incubated at 30 °C for 24 h in a 250 mL flask containing 50 mL of LB (tryptone 10 g/L, yeast extract 5 g/L, NaCl 10 g/L). The resulting cultures were then transferred into fresh LB for further incubation at 30 °C. Until the optical density at 600 nm (OD_600_) reached 0.6, bacterial cells were collected and washed three times with sterile water to remove residual medium. Subsequently, the cells were incubated in 100 mL of MSM containing PVC powders as the sole carbon source at 30 °C for 60 days. Uninoculated medium containing PVC powder (abiotic control) and inoculated medium without PVC powders (PVC-free control) were prepared under the same conditions. The growth curves of strains were monitored by measuring OD_600_ using a spectrophotometer (Shanghai Sunny Hengping Scientific Instrument Co., Ltd., Shanghai, China) over a 42-day period. To measure the OD600 of the experimental group (inoculated medium containing PVC powder) and PVC-free control, the abiotic control and uninoculated PVC-free MSM control (uninoculated medium without PVC powder) were used as the blanks for zeroing the spectrophotometer, respectively. All experiments were performed in triplicate. After 60 days of incubation, PVC powders were washed with sterile water to remove cells attached to their surface. The powders were then treated with 2% (*w*/*v*) sodium dodecyl sulfate (SDS) and ultrasonicated for 3 min to remove the biofilms. Subsequently, the residual SDS was fully removed by washing the powders with sterile water. Finally, the powders were treated with absolute ethanol to remove the residual water. Each of these treatments were performed three times. Treated powders were air-dried and analyzed by ATR-FTIR. During ATR-FTIR analysis, the powders were scanned 64 times in the wavenumber range of 4000–400 cm^−1^ using an FTIR spectrometer (Nicolet iS5, Thermo Fisher Scientific, Waltham, MA, USA).

### 2.3. 16S rRNA Gene Sequencing

For genus identification, the strain was incubated in 3 mL of LB at 30 °C for 12 h. After incubation, the bacterial cells were harvested by centrifugation at 12,000 rpm for 1 min, and then resuspended in sterile water. The suspension was incubated at 100 °C for 10 min, and then centrifuged at 12,000 rpm for 10 min. After centrifugation, the supernatant was collected and used as the genomic DNA template for PCR. The universal primers 27F (5′-AGAGTTTGATCCTGGCTCAG-3′) and 1492R (5′-GGTTACCTTGTTACGACTT-3′) were used for polymerase chain reaction (PCR) to amplify the 16S rRNA gene. The PCR was performed using a thermal cycler (Bio-Rad, Hercules, CA, USA) The PCR mixture (50 μL) consisted of 0.4 μM each primer, 10 μL of 5× PrimeSTAR buffer, 200 μM dNTPs, 50 ng of genomic DNA, 1.25 U of PrimeSTAR HS DNA polymerase (Takara Biotechnology (Dalian) Co., Ltd., Dalian, China), 2 μL of dimethyl sulfoxide, and sterile water to 50 μL. The PCR thermal cycle included an initial denaturation at 98 °C for 10 min, followed by 30 cycles of 98 °C for 10 s, 55 °C for 5 s, and 72 °C for 1.5 min. A final extension step was performed at 72 °C for 10 min. The PCR products were sent to General Biol (Anhui) Co., Ltd. (Chuzhou, China) for 16S rRNA sequencing. The 16S rRNA gene sequence was analyzed by BLASTn (version 2.17.0+) online (https://blast.ncbi.nlm.nih.gov/Blast.cgi, accessed on 21 September 2026) and deposited in the GenBank database. MEGA7 (version 7.0.18) software was employed for phylogenetic analysis [22]. The phylogenetic tree was constructed by the neighbor-joining method with 1000 bootstrap replicates.

### 2.4. Whole-Genome Sequencing of Strain

To identify the strain at the genomic level and genes encoding potential enzymes involved in PVC biodegradation pathway, whole-genome sequencing of this strain was performed. The strain was cultured in a 250 mL flask at 30 °C for 24 h. After cultivation, bacterial cells were harvested from the culture and sent to Wuhan Benagen Technology Co., Ltd. (Wuhan, China) for whole-genome sequencing. Illumina and Nanopore platform were used for genome sequencing, with a coverage depth of 486× and 305×, respectively [23]. The reads were assembled into contigs using Unicycler (version 0.5.0) [24]. The quality of the assembled genome, including genome completeness and potential contamination, was systematically evaluated using CheckM2 (version 1.1.0) [25]. The average nucleotide identity (ANI) and digital DNA-DNA hybridization (dDDH) values between the target strain and its closely related type strains were calculated using the ANI Calculator (https://www.ezbiocloud.net/tools/ani, accessed on 20 September 2026) [26] and the GGDC web server (https://ggdc.dsmz.de/ggdc.php, accessed on 20 September 2026) [27]. Functional annotation of genes was performed using Pfam (version 37.0) [28], KEGG (https://www.kegg.jp/kegg/, accessed on 23 July 2026) [29], COG (http://www.ncbi.nlm.nih.gov/COG, accessed on 23 July 2026) [30], GO (http://geneontology.org/, accessed on 23 July 2026) [31], UniProt (https://www.uniprot.org/, accessed on 23 July 2026) [32], and CAZy (http://www.cazy.org/, accessed on 23 July 2026) [33] databases. MEGA7 (version 7.0.18) was used for phylogenetic analysis of laccases, catalase-peroxidases, and dehalogenases.

### 2.5. Expression and Purification of Catalase-Peroxidase

The gene *cat-3317* derived from the strain was synthesized by Beijing Tsingke Biotech Co., Ltd. (Beijing, China) and ligated into pET-28a (+) vector using the *Nde*I and *Bam*HI restriction sites. The recombinant vector was transformed into *Escherichia coli* (*E. coli*) BL21 (DE3) for protein expression. For expression of recombinant protein, cells harboring the recombinant vector were inoculated into 5 mL of LB containing 30 μg/mL kanamycin and incubated overnight at 37 °C and 220 rpm. The culture was transferred into 500 mL fresh LB containing kanamycin and incubated at 37 °C until the OD_600_ reached 0.4 to 0.6. Recombinant protein expression was induced with isopropyl β-D-1-thiogalactopyranoside (IPTG) at a final concentration of 0.4 mM at 16 °C for 24 h. Hemin (final concentration of 0.02 mM) was added for fully active peroxidase production. After cultivation, cells were harvested by centrifugation at 5000 rpm and 4 °C for 5 min. Harvested cells were resuspended in lysis buffer (NaH_2_PO_4_ 50 mM, NaCl 300 mM, imidazole 10 mM, pH 8.0) and then disrupted by sonication using an ultrasonic homogenizer (JY92-IIN, Ningbo Scientz Biotechnology Co., Ltd., Ningbo, China). The crude enzyme was obtained by centrifuging the cell suspension at 12,000 rpm and 4 °C for 20 min. The crude enzyme was purified using a Ni-NTA column. The purified recombinant protein was eluted with elution buffer (NaH_2_PO_4_ 50 mM, NaCl 300 mM, imidazole 250 mM, pH 8.0). Afterwards, salts and imidazole in enzyme solution were replaced with 50 mM Tris-HCl (pH 8.0) using a desalting column. The desalted protein was detected by sodium dodecyl sulfate-polyacrylamide gel electrophoresis (SDS-PAGE) and used for subsequent experiments. Concentration of the desalted protein was measured by Bradford method. The UV-Vis spectrum of recombinant protein was measured using a spectrophotometer (UV-2600, Shimadzu, Kyoto, Japan).

### 2.6. Catalase-Peroxidase Assay

To determine the optimal reaction conditions of the catalase-peroxidase, 2,6-dimethoxyphenol (DMP, ε469 = 27,500 M^−1^ cm^−1^) was used as the substrate. In all enzymatic assays, the reaction was initiated by the addition of H_2_O_2_ to a final concentration of 1 mM. To determine the optimal temperature, enzyme reactions were performed at different temperatures from 5 to 50 °C in 100 mM Tris-HCl buffer (pH 8.0). The optimal pH was determined using buffers (50 mM) with different pH levels, including citrate-phosphate buffer (pH 3.0–8.0), Tris-HCl buffer (pH 8.0–9.0) and glycine-sodium hydroxide buffer (pH 9.0–10.6) at the optimal temperature. Temperatures of 20 °C and 30 °C were selected for thermostability assays. The enzyme was preincubated at 20 °C and 30 °C for 6 h, 17 h, 22 h, 27 h, and 40 h. Subsequently, the treated-enzyme were cooled on ice and added to the reaction systems. To evaluate the stability of the enzyme at pH 8.0 and 9.0 at 30 °C, proteins were incubated in Tris-HCl buffer for 6 h, 17 h, 22 h, 27 h, 40 h, 48 h, and 72 h, respectively. During the stability assays, untreated and boiling-inactivated proteins were used as the positive and negative controls, respectively. One unit of activity was defined as the amount of enzyme required to oxidize 1 μmol of substrate per minute. Reaction kinetics were tested at the optimal pH and temperature, and the concentration of substrate ranged from 0.125 mM to 5 mM. All the above experiments were performed in triplicate.

### 2.7. PVC Degradation Assay of Catalase-Peroxidase

To evaluate the PVC-degrading ability of the catalase-peroxidase, additive-free PVC film was prepared as described in a previous study [21]. PVC powder (0.6 g) was added to 30 mL of tetrahydrofuran (Tianjin De’en Chemical Reagents Co., Ltd., Tianjin, China), and the mixture was stirred continuously to obtain a transparent solution. This solution was poured into a glass plate, and the solvent was evaporated as slowly as possible. The resulting films were cut into pieces (1 cm × 1 cm) and cleaned with sterile water and 75% (*v*/*v*) ethanol. The reaction mixture (5 mL) used for the degradation assay contained 50 μg/mL purified enzyme [13], 1 mM H_2_O_2_, and 5 pieces of PVC film in 50 mM Tris-HCl buffer (pH 8.0). The control group had the same composition as the experimental group, except that the heat-inactivated enzyme was used instead of the untreated enzyme. The degradation reaction was performed for 96 h in a shaker (150 rpm, 30 °C). Fresh enzyme and 1 mM H_2_O_2_ were added if needed. After incubation, films were cleaned as described above and analyzed by ATR-FTIR, GPC (1260, Agilent Technologies, Santa Clara, CA, USA), and SEM (sigma 360, Cari Zeiss, Oberkochen, Germany). The reaction mixture was centrifuged and the supernatant was collected. The supernatant was freeze-dried and re-dissolved in dichloromethane (Tianjin De’en Chemical Reagents Co., Ltd., Tianjin, China). The degradation products were analyzed by GC-MS (TQ8040, Shimadzu, Kyoto, Japan). The compounds were separated using an SH-Rxi-5Sil MS column (30 m × 0.25 mm × 0.25 μm). Both interface and ion source temperatures were 280 °C. Electron impact mass spectra were recorded in the 60–600 *m*/*z* mass range under 70 eV ionization energy. The scan time was 0.2 s. Helium was used as carrier gas under constant linear velocity control mode. The average linear velocity was set to 49.2 cm/s, corresponding to a column flow rate of 1.82 mL/min. The purge flow was maintained at 3 mL/min, and the split ratio was 3.0:1. The oven temperature program was initially held at 60 °C for 2 min, then increased at a rate of 20 °C/min to 280 °C and held for 3 min. Degradation products were identified according to the NIST mass spectral database.

## 3. Results

### 3.1. Enrichment Culture and Isolation of PVC-Degrading Strain from Soil

After enrichment culture, 20 colonies were individually picked and inoculated into MSM with 1% (*w*/*v*) PVC powder as the sole carbon source. After incubation for 60 days, two of the 20 colonies were able to grow. The strain exhibiting the highest OD_600_ value was designated PVC-3-16 and selected for further study.

**Figure 1:**
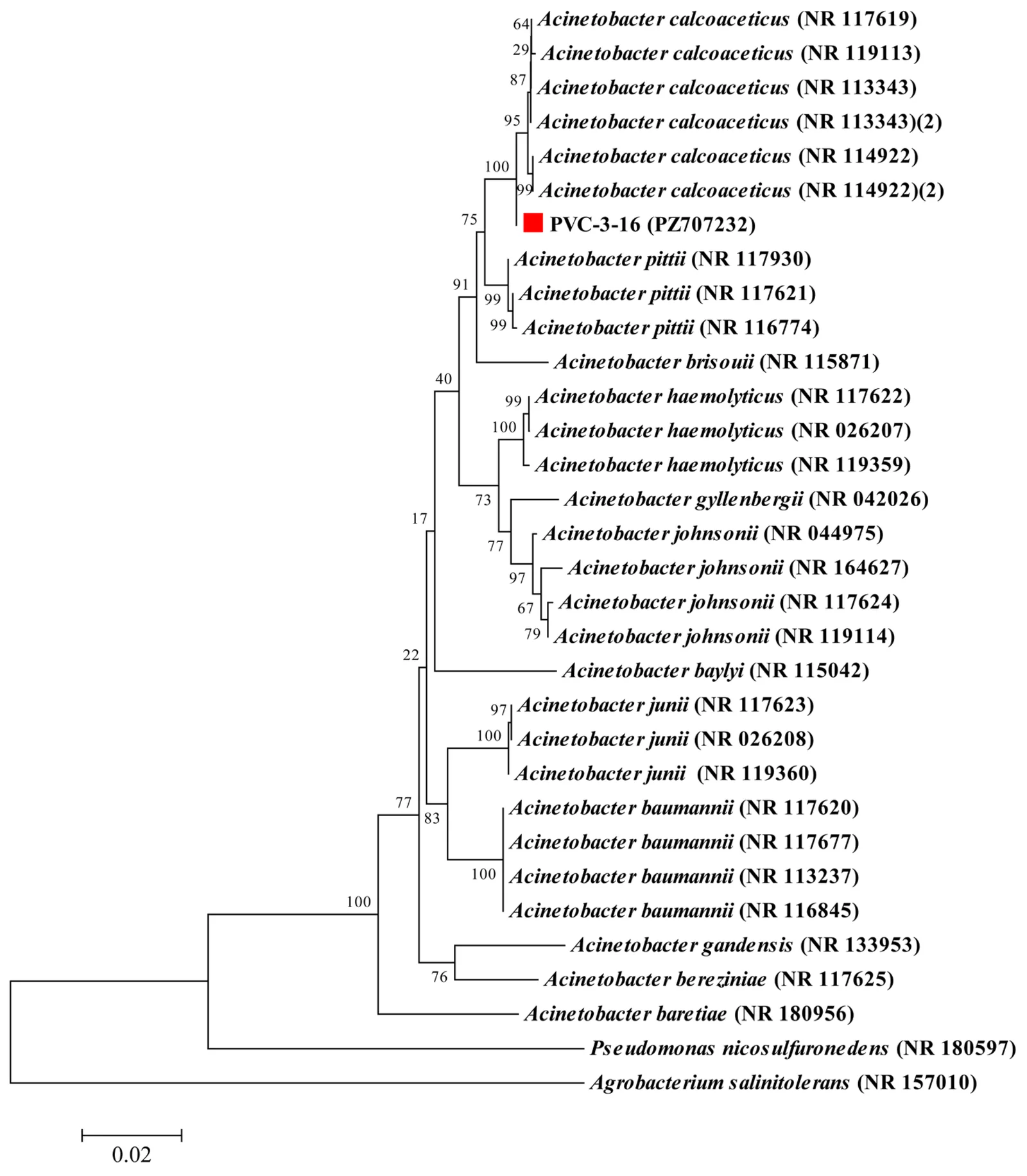
Phylogenetic analysis of strain PVC-3-16 based on 16S rRNA gene sequences.

The 16S rRNA gene sequence of strain PVC-3-16 was deposited in GenBank under accession number PZ707232. BLASTn (https://blast.ncbi.nlm.nih.gov/Blast.cgi, accessed on 21 September 2026) analysis showed that the 16S rRNA gene sequence of PVC-3-16 shared the highest identity (99.71%) with that of *Acinetobacter calcoaceticus* ATCC 23055 under accession number NR_117619. Figure 1 shows the phylogenetic tree of strain PVC-3-16 based on the 16S rRNA gene sequence. This indicated that PVC-3-16 belonged to the genus *Acinetobacter*.

### 3.2. PVC Biodegradation Assay

**Figure 2:**
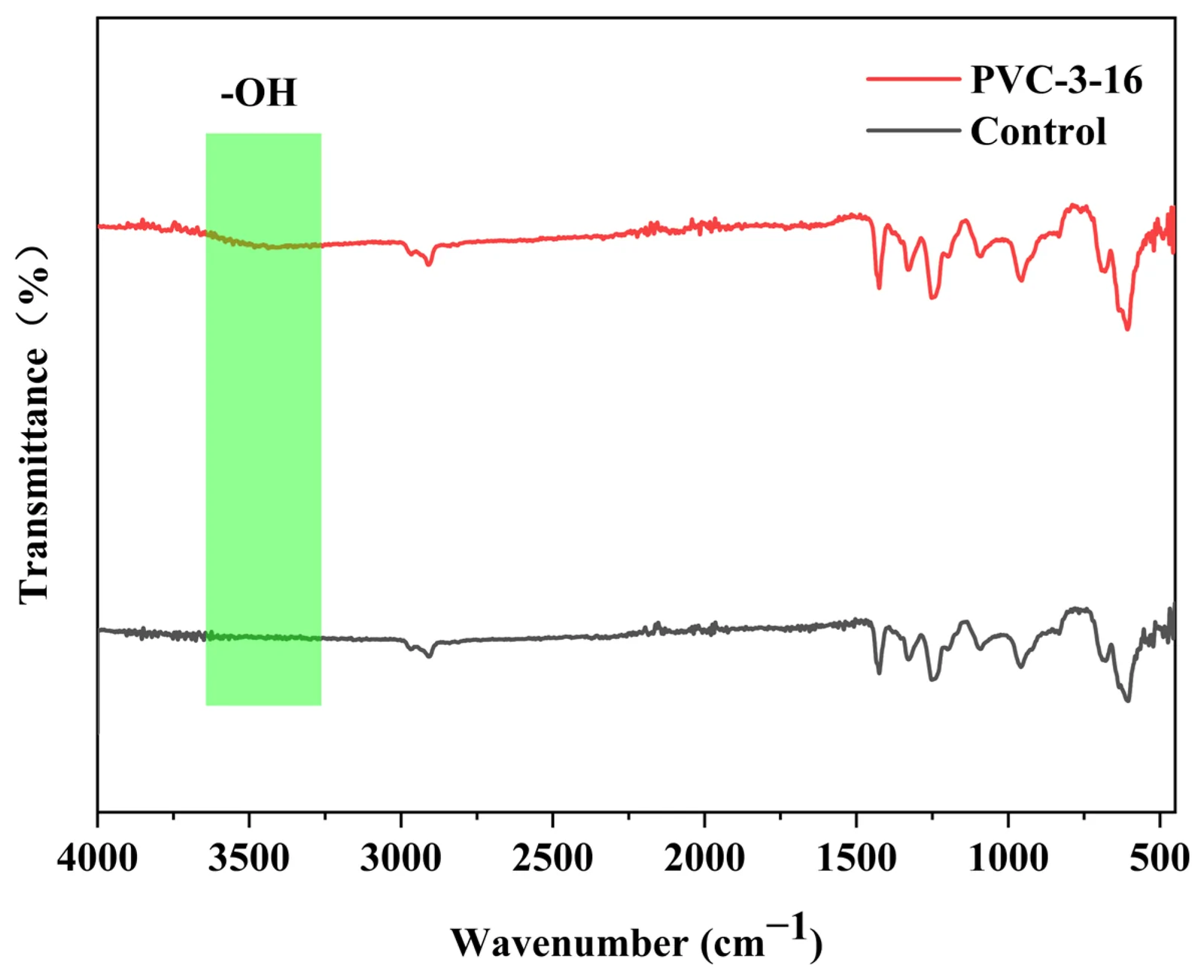
ATR-FTIR spectra of treated and untreated PVC powders.

As shown in Figure S1, after 42 days incubation, the OD_600_ value of experimental group was 0.096 higher than that of PVC-free control. The growth curve of PVC-3-16 indicated that PVC-3-16 entered the stationary phase after incubation for 42 days. As shown in Figure 2, the ATR-FTIR spectra of PVC-powders treated with PVC-3-16 showed a new peak in the region of 3300–3500 cm^−1^ compared with the control group. This indicated the emergence of new functional groups (-OH) on the surface of PVC powders after incubation with PVC-3-16.

### 3.3. Genomic Data Mining for PVC Degrading Enzymes

To further identify the enzymes involved in PVC degradation, whole-genome sequencing was performed. The sequencing assembly yielded a single circular contig of 4,003,094 bp. The completeness and contamination were 100% and 0.12%, respectively. The genomic data of strain PVC-3-16 were deposited in the NCBI database under accession number NZ_CM188126.1. The GC content of the PVC-3-16 genome was 38.76%. Figure S2 shows the whole-genome circular map of PVC-3-16. The genome sequence of PVC-3-16 shared the highest ANI (96.09%) and dDDH (83.7%) values with *Acinetobacter calcoaceticus* (accession number NZ_UFSJ01000001), indicating that PVC-3-16 belongs *Acinetobacter calcoaceticus*. As shown in Figure S3, the result of KEGG-based annotation indicated that 137 genes were associated with xenobiotic biodegradation and metabolism in the genome of PVC-3-16.

Several genes encoding plastic-degrading enzymes, such as laccases, monooxygenases, catalase-peroxidases, and dehalogenases, were identified in the genome of strain PVC-3-16. Among them, a putative catalase-peroxidase (accession number WP_500968708.1) shared the highest identity (95%) and similarity (97%) with a PVC-degrading enzyme (accession number XXC75028.1) was designated CAT-3317. Furthermore, a putative laccase (accession number WP_500968615.1) shared the highest identity (93%) and similarity (95%) with a PVC-degrading enzyme (accession number XXC75246.1) was designated LAC-3071. The theoretical molecular weights of CAT-3317 and LAC-3017 predicted by ExPASy were 78.6 kDa and 70.1 kDa, respectively [34].

The Pfam database was used to analyze the genomic data of PVC-3-16. The results showed the presence of 17 dehalogenase-related genes in the genome of PVC-3-16. Furthermore, a putative phosphoserine phosphatase (designated as PSP-3) with the protein accession number WP_500967200.1 was selected for analysis. The amino acid sequence of this enzyme shared 39% identity and 60% similarity with the characterized phosphoserine phosphatase (SerB), which catalyzed dechlorination reaction in the PVC degradation pathway [35]. The phylogenetic analysis of putative dehalogenases is shown in Figure S4.

### 3.4. Catalase-Peroxidase Enzyme Assay

After desalting, the recombinant protein CAT-3317 exhibited a reddish-brown color. As shown in Figure S5A, the UV-Vis spectrum of recombinant protein showed a Soret band at 408 nm. The Reinheitszahl (Rz) value (A_408_/A_280_) of the recombinant protein was 0.79, indicating proper binding of hemin in the active site. A single distinct band corresponding to the expected 78.6 kDa protein was observed in Figure S5B, migrating immediately above the 72 kDa marker band, well separated from the 95 kDa marker band.

**Figure 3:**
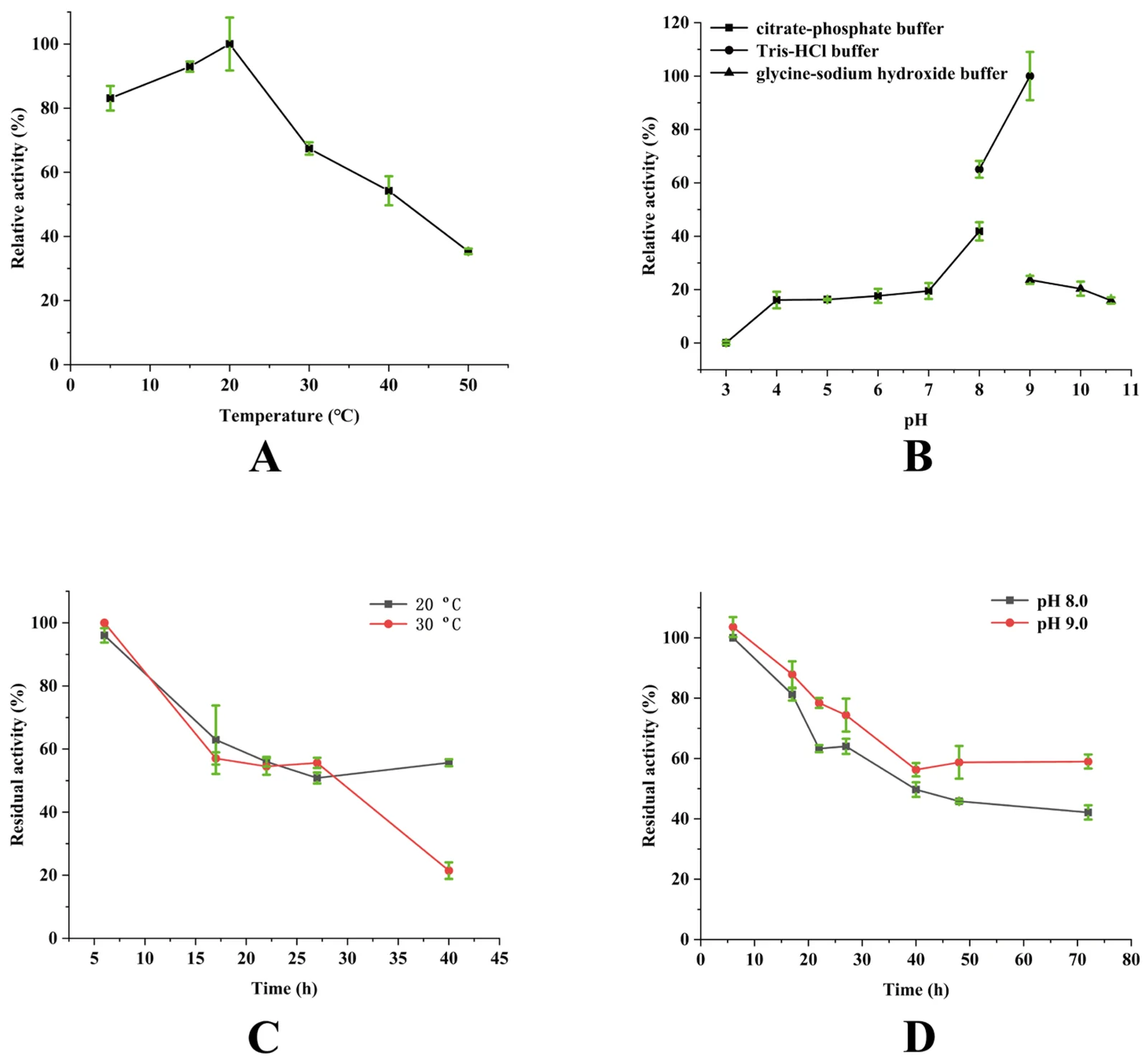
Effect of pH and temperature on the activity of CAT-3317: (**A**) optimal temperature; (**B**) optimal pH; (**C**) thermal stability at 20 °C and 30 °C; (**D**) pH stability at pH 8.0 and pH 9.0 in Tris-HCl buffer.

The optimal temperature and pH of CAT-3317 were determined using DMP as the substrate. As shown in Figure 3A, the optimal temperature was 20 °C. At the other tested temperatures, CAT-3317 retained more than 50% of its maximum activity, except at 50 °C. In particular, the enzyme retained more than 80% of its maximum activity at 5 °C. As shown in Figure 3B, the optimal pH was 9.0 (Tris-HCl buffer). CAT-3317 retained less than 50% and 30% of its maximum activity in citrate-phosphate buffer (pH 3.0–8.0) and glycine-sodium hydroxide buffer (pH 9.0–10.6), respectively. The thermostability of the enzyme is shown in Figure 3C. After incubation at 20 °C and 30 °C for 27 h, the enzyme retained more than 50% of its activity. After incubation at 20 °C and 30 °C for 40 h, the residual activity of the enzyme was 55% and 22%, respectively. As shown in Figure 3D, the enzyme retained more than 50% of its initial activity after incubation at pH 8.0 for 40 h and at pH 9.0 for 72 h. The *K_m_*, *k_cat_*, and *k_cat_*/*K_m_* values of CAT-3317 toward DMP were 2.72 ± 0.49 mM, 7.24 ± 0.89 s^−1^, and 2.68 ± 0.23 s^−1^·mM^−1^, respectively. The specific activity towards DMP was 3.43 ± 0.13 U/mg.

### 3.5. Assessment of PVC-Degrading Ability of Catalase-Peroxidase

**Figure 4:**
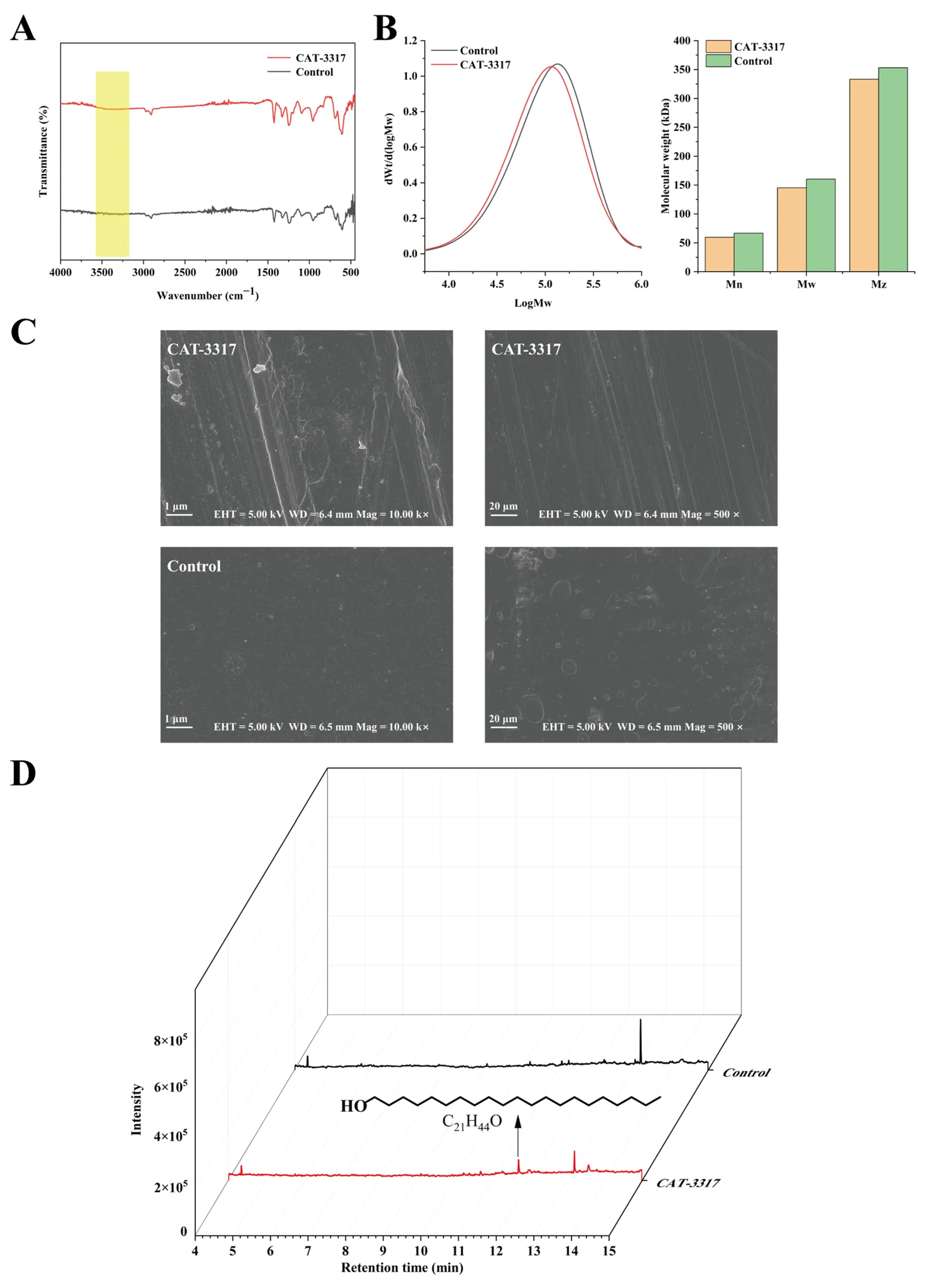
Physicochemical characterization of PVC films treated with CAT-3317: (**A**) ATR-FTIR; (**B**) GPC; and (**C**) SEM results of treated film; (**D**) the result of GC-MS of degradation product catalyzed by CAT-3317.

The ATR-FTIR spectra of enzyme-treated group revealed a new peak at 3451 cm^−1^, consistent with O–H strectching, compared with those of the heat-inactivated negative control group. The PVC films incubated with CAT-3317 showed reductions of 10.7%, 9.4%, and 5.7% in M_n_, M_w_, and M_z_ values, respectively. The SEM results revealed that incubation with CAT-3317 resulted in cracks on the surface of PVC films. Using GC-MS, a potential degradation product with a retention time of 11.7 min was identified as C_21_H_44_O. All the results mentioned above are shown in Figure 4, and the mass spectrum is shown in Figure S6.

## 4. Discussion

Plastic products, especially PVC, are widely used, causing serious environmental pollution. Biodegradation of PVC has drawn significant attention from researchers because it is environmentally friendly. In previous studies, several microorganisms with PVC-degrading ability have been identified [12,13,14,21,36]. However, PVC-degrading enzymes have rarely been characterized. In this study, a strain of *Acinetobacter calcoaceticus* designated PVC-3-16 was isolated from farmland soil. Its genome was analyzed and a catalase-peroxidase exhibiting PVC-degrading ability was characterized.

The strain PVC-3-16 could grow in MSM containing PVC as the sole carbon source. Its PVC-degrading ability was further supported by ATR-FTIR analysis. Peng et al. reported that the formation of carbonyl and hydroxyl groups is a prerequisite for both oxidation and biodegradation of PVC [37]. In this study, the FTIR spectra revealed the presence of hydroxyl groups, and carbonyl groups were not detected. Zhang et al. observed hydroxyl groups and carbon–carbon double bonds on the surface of PVC film after incubation with the EMBL-1 strain for 90 days [13], whereas only hydroxyl groups were detected in another study using strain PVC-6A [21]. Meanwhile, Giacomucci et al. reported that neither carbonyl nor hydroxyl groups were detected, and changes in the FTIR spectrum occurred only between 800 cm^−1^ and 1500 cm^−1^ [12]. In contrast, the FTIR spectrum of PVC treated with *Pseudomonas aeruginosa* showed a decrease only in the intensity of the C-Cl stretching peak [36]. These results suggest that the initial attack on PVC by strain PVC-3-16 occurs mainly through hydroxylation.

Depolymerization and dechlorination are considered to be key steps in PVC biodegradation pathway. In previous studies, catalase-peroxidases and laccases have been identified as the enzymes involved in the depolymerization and dechlorination of PVC [13,21]. Due to the highest sequence identity and similarity with previously reported PVC-degrading enzymes, CAT-3317 and LAC-3071 were identified as candidate PVC-degrading enzymes. In addition, dehalogenases are necessary for the dechlorination reaction in the PVC degradation pathway. Genomic analysis of PVC-3-16 revealed that all the putative dehalogenases in this strain belonged to the HAD superfamily. SerB has been proven to catalyze the dechlorination process during PVC degradation [35]. As shown in Figure S4, the phylogenetic analysis showed that PSP-3 clustered with SerB in the same clade. These results suggest that PSP-3 plays a potential role in the dechlorination step of PVC biodegradation.

According to previous studies, the depolymerization and dechlorination products of PVC would be further degraded by monooxygenase, alcohol dehydrogenase, esterase, dioxygenase, and aldehyde dehydrogenase. The final degradation products of enzymatic catalysis are then metabolized through β-oxidation. The oxidation product is transferred into the tricarboxylic acid (TCA) cycle to support microbial metabolic processes [13]. Genes related to all of the above-mentioned enzymes can be found in the genomic data of strain PVC-3-16.

To confirm the PVC-degrading ability of CAT-3317, the enzyme was successfully expressed and purified. The enzyme retained most of its activity at 5 °C, indicating that it is a cold-adapted catalase-peroxidase. However, it was more stable at 20 °C (the optimal temperature) than at 30 °C. To match the growth temperature of PVC-3-16, 30 °C was selected to perform the PVC-degrading assay. In a previous study, a catalase-peroxidase performed the depolymerization reaction at 30 °C [13]. In another study, although the optimal temperature of a laccase derived from *Lysinibacillus fusiformis* was 70 °C, the PE degradation reaction was carried out at 30 °C [19]. In the present study, the optimal pH of CAT-3317 was 9.0 in Tris-HCl buffer, and it retained more than 65% of its maximum activity at pH 8.0 (Tris-HCl buffer). However, at pH 8.0 (citrate-phosphate buffer) and pH 9.0 (glycine-sodium hydroxide buffer), it retained only 40% and 23% of its maximum activity. These results indicated that CAT-3317 showed higher activity in Tris-HCl buffer than in citrate-phosphate buffer or glycine-sodium hydroxide buffer. Considering the stability of H_2_O_2_ under alkaline conditions, pH 8.0 was selected for the catalase-peroxidase-mediated PVC degradation assay, although CAT-3317 was more stable at pH 9.0 than at pH 8.0. This approach is consistent with a previous study in which a laccase-mediated PE degradation assay was carried out at pH 7.0 rather than at the optimal pH (3.0) of the laccase [38].

The ATR-FTIR results of the catalase-peroxidase-mediated PVC degradation assay showed the formation of hydroxyl groups on the surface of PVC film, indicating the occurrence of chemical reactions involving PVC. This is consistent with the results of a previous study [21]. The depolymerization of plastics can be classified into two categories: (1) limited-extent depolymerization, in which M_n_ alone or both M_n_ and M_w_ increase; and (2) broad depolymerization, in which both M_n_ and M_w_ decrease [39]. The GPC results showed that the M_n_, M_w_, and M_z_ values of PVC decreased after treatment with CAT-3317 compared with those of the negative control group. These results indicated that CAT-3317 caused broad depolymerization of PVC. GPC analysis further showed an increase in fragments with medium molecular weights. These results indicated that CAT-3317 catalyzed the depolymerization reaction to form fragments with medium molecular weight. The degradation product generated by CAT-3317 was detected by GC-MS and identified as C_21_H_44_O, suggesting that CAT-3317 catalyzed PVC depolymerization accompanied by dechlorination of PVC. In previous studies, GC-MS analysis showed that the degradation products generated by catalase-peroxidases were identified as long-chain alkanes (C_20_H_42_, C_21_H_44_, C_24_H_50_, and C_25_H_52_) [13] and long-chain fatty acids (C_16_H_32_O_2_ and C_18_H_36_O_2_) [21]. No degradation products containing carbon–carbon double bonds were detected, either in the present study or in the previous studies. These results indicate that catalase-peroxidases mediate the dechlorination of PVC with an unknown mechanism.

In conclusion, a strain with PVC-degrading ability was isolated from farmland and designated PVC-3-16. This strain could efficiently utilize PVC as the sole carbon source for growth. The PVC-degrading ability of PVC-3-16 was further supported by ATR-FTIR. The results of 16S rRNA, ANI, and dDDH analyses indicated that the strain belonged to *Acinetobacter calcoaceticus*. A putative catalase-peroxidase derived from the genome of PVC-3-16 was expressed in *E. coli*. Typical degradation features, such as cracks and the occurrence of hydroxyl groups, were detected on the surface of films after treatment with CAT-3317. Compared with the control group, the catalase-peroxidase-mediated depolymerization resulted in decreases of 10.7%, 9.4%, and 5.7% in M_n_, M_w_, and M_z_ values, respectively. A primary alcohol degradation product of PVC was identified by GC-MS after treatment with the catalase-peroxidase. This study provides an important reference for the isolation and identification of PVC-degrading bacteria and offers deep insights into the mechanism of PVC biodegradation.

## Data Availability

All essential data in this study such as the accession numbers of genome and enzyme, whole-genome circular map of strain and mass spectrum are included in the article and Supplementary Materials. The raw data (such as GPC and GC-MS) supporting this ar-ticle are available from the corresponding author upon request.

## Supplementary Materials

The following supporting information can be downloaded at https://www.mdpi.com/article/10.3390/microorganisms14102332/s1, Figure S1: The grow curve of strain PVC-3-16.; Figure S2: The whole-genome circular map of strain PVC-3-16; Figure S3: KEGG metabolic pathway analysis of strain PVC-3-16; Figure S4: The phylogenetic analysis of dehalogenases derived from strain PVC-3-16; Figure S5: UV-Vis spectrum of CAT-3317 and SDS-PAGE analysis of CAT-3317; Figure S6: The mass spectrum and structural formula of the potential degradation product.

## Abbreviations

The following abbreviations are used in this manuscript:

PVC	Polyvinyl chloride
PE	Polyethylene
PU	Polyurethane
PP	Polypropylene
PET	Polyethylene terephthalate
PS	Polystyrene
MSM	Mineral salt medium
LB	Luria broth
SDS	Sodium dodecyl sulfate
ATR-FTIR	Attenuated total reflection Fourier transform infrared spectroscopy
SEM	Scanning electron microscope
GPC	Gel permeation chromatography
GC-MS	Gas chromatography-mass spectrometry
ANI	Average nucleotide identity
dDDH	Digital DNA-DNA hybridization
IPTG	Isopropyl β-D-1-thiogalactopyranoside
