# Supplementary material for: Biodegradation of Polyvinyl Chloride by Acinetobacter sp. Isolated from Soil
Source: Microorganisms. 2026 Oct 13;14(10):2332. doi: 10.3390/microorganisms14102332 (PMC13707438; doi:10.3390/microorganisms14102332)
Supplement: Supplementary file 1 [file microorganisms-14-02332-s001.zip › supplementary material.pdf]

Biodegradation of Polyvinyl Chloride by *Acinetobacter* sp. Isolated from Soil

Shumao Chai [1,*], Jiahao Wang [1], Yiru Zhang [1], Zihan Zhang [1], Zihan Yang [1], Yitong Wei [1] and Mingfu Niu [2]

[1] College of Food and Drug, Luoyang Normal University, Luoyang 471934, China; 232364003@lynu.edu.cn (J.W.); 252382102@lynu.edu.cn (Y.Z.); 252354014@lynu.edu.cn (Z.Z.); 252354046@lynu.edu.cn (Z.Y.); 252354024@lynu.edu.cn (Y.W.)
[2] College of Food and Bioengineering, Henan University of Science and Technology, Luoyang 471023, China; mingfuniu@163.com
* Correspondence: chaishumao@lynu.edu.cn

Abstract

The excessive use of plastics has seriously damaged the environment. Biodegradation of plastics, especially polyvinyl chloride (PVC), by microorganisms has become a promising strategy for the remediation of environmental pollution. Plastic-contaminated soil is an important source for isolating microorganisms with plastic-degrading ability. In this study, a PVC-degrading strain, designated PVC-3-16, was isolated from farmland soil and identified as *Acinetobacter calcoaceticus*. After 60 days of incubation, a new characteristic functional group was detected on the surface of PVC powder by attenuated total reflection Fourier transform infrared spectroscopy. A putative catalase-peroxidase enzyme derived from the genome of PVC-3-16 was successfully expressed and purified. After treatment with the catalase-peroxidase, hydroxyl groups and cracks were detected on the surface of PVC films by attenuated total reflection Fourier transform infrared spectroscopy and scanning electron microscopy, respectively. The M_n, M_w, and M_z values of the PVC films decreased by 10.7%, 9.4%, and 5.7%, respectively, after treatment. The degradation product catalyzed by the catalase-peroxidase was identified as $C_{21}H_{44}O$. These findings indicate that PVC-3-16 is a promising strain that can be used for PVC biodegradation.

Keywords: polyvinyl chloride; biodegradation; *Acinetobacter*; catalase-peroxidase

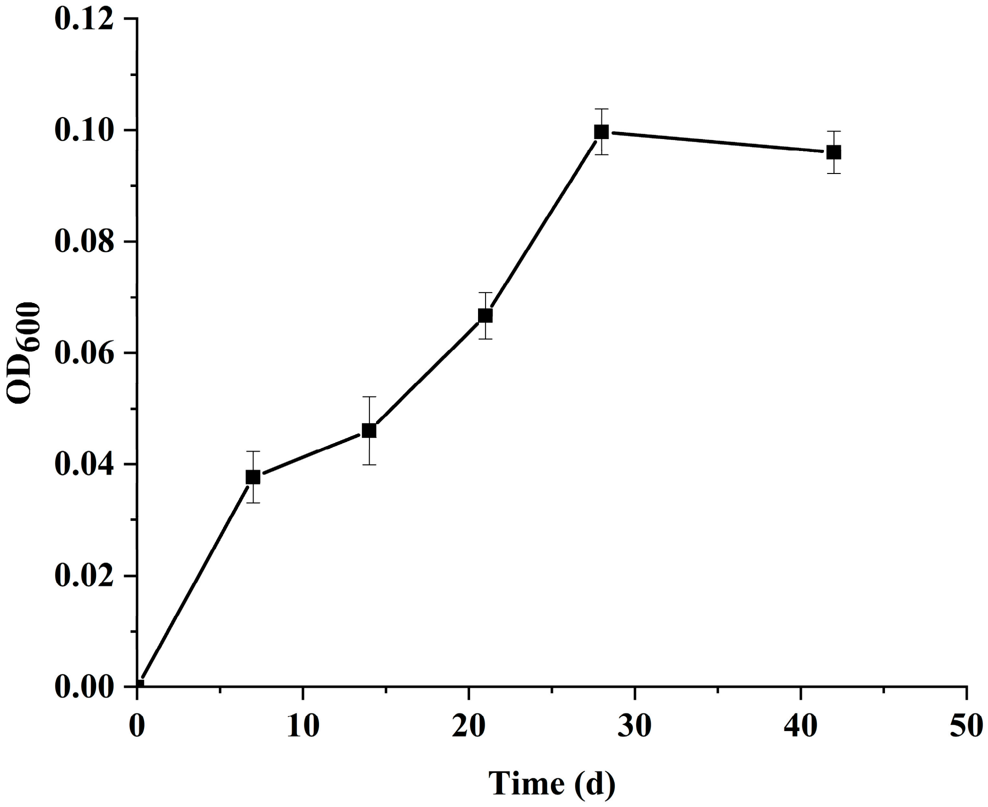

Figure S1. The grow curve of strain PVC-3-16.

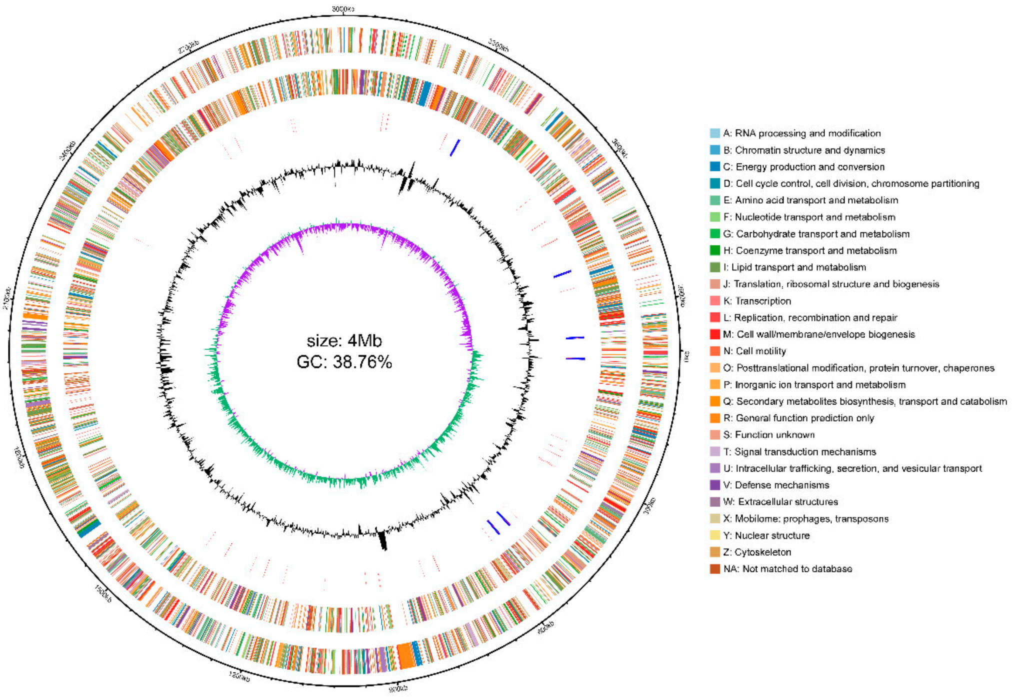

Figure S2. The whole-genome circular map of strain PVC-3-16.

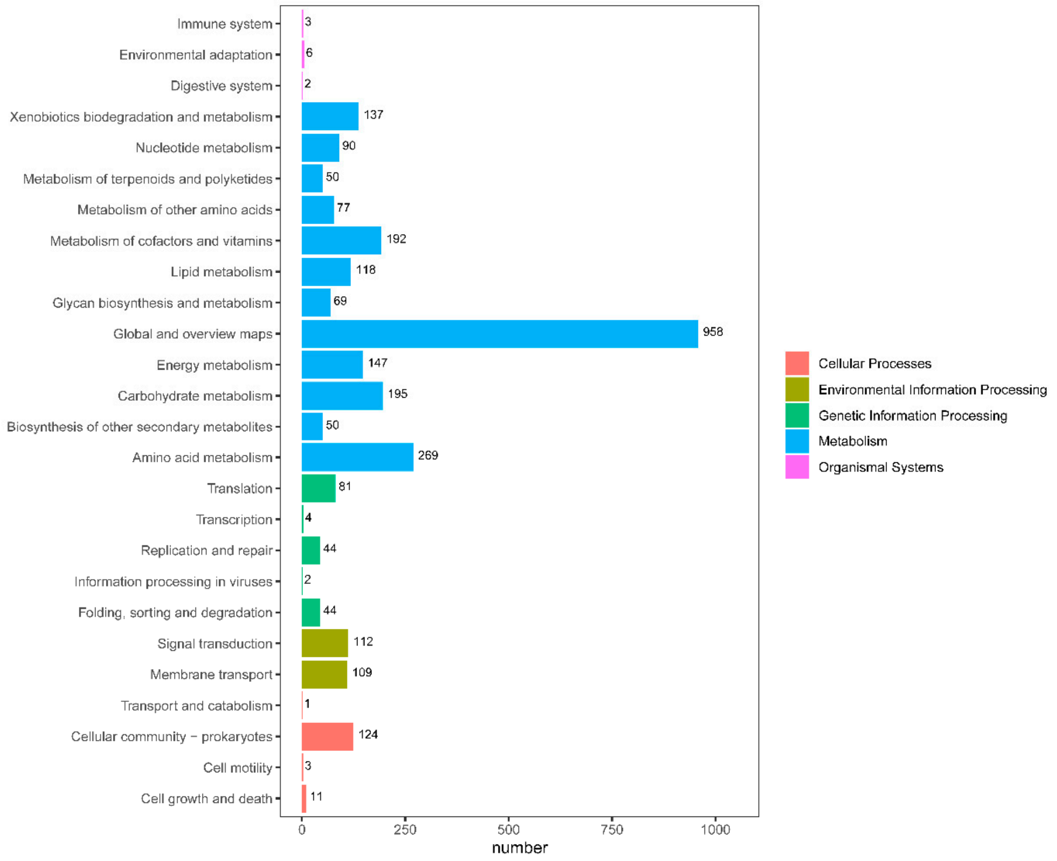

Figure S3. KEGG metabolic pathway analysis of strain PVC-3-16. The genes involved in the metabolic pathway were classified to five branches. Cellular Processes, Environmental Information Processing, Genetic Information Processing, Metabolism and Organismal Systems were marked with orange, grayish green, green, blue and purple, respectively.

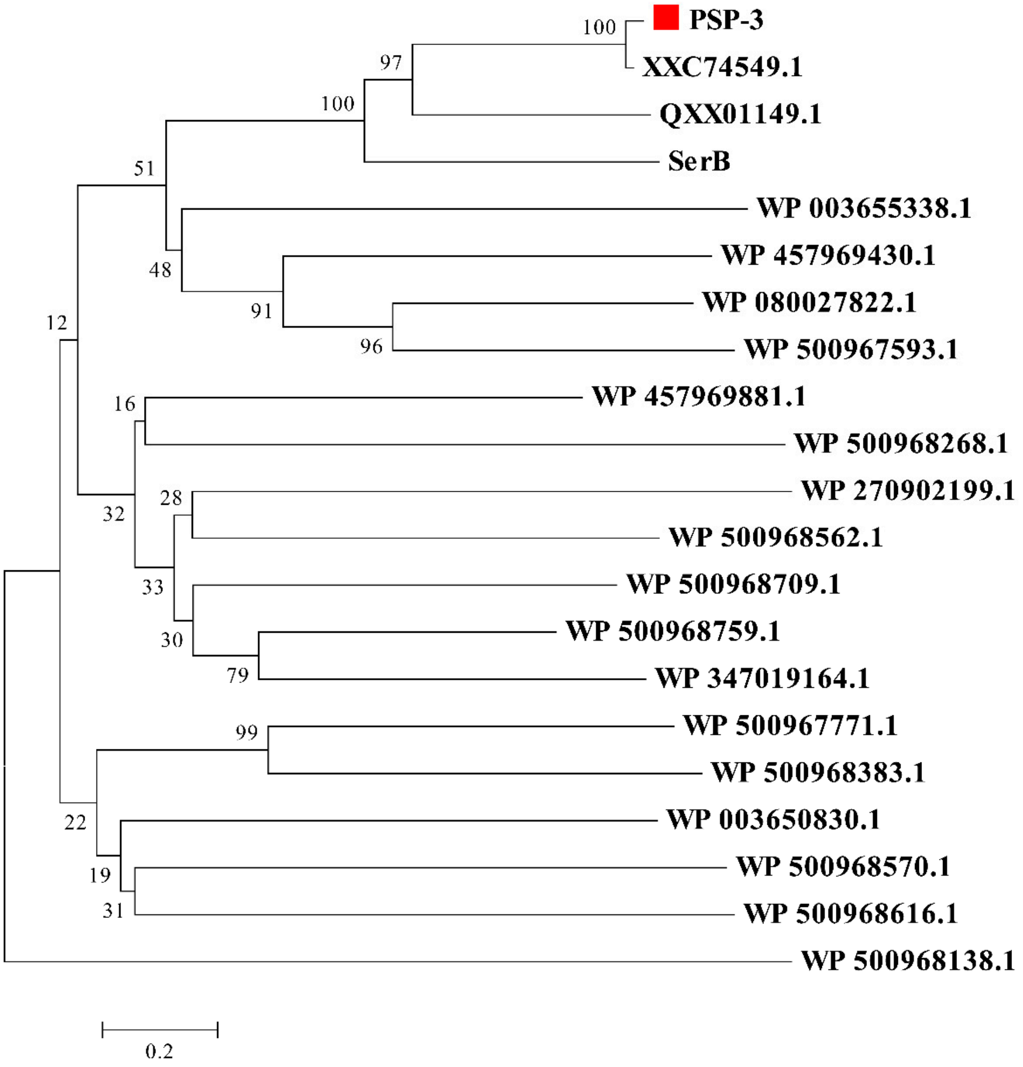

Figure S4. The phylogenetic analysis of dehalogenases derived from strain PVC-3-16.

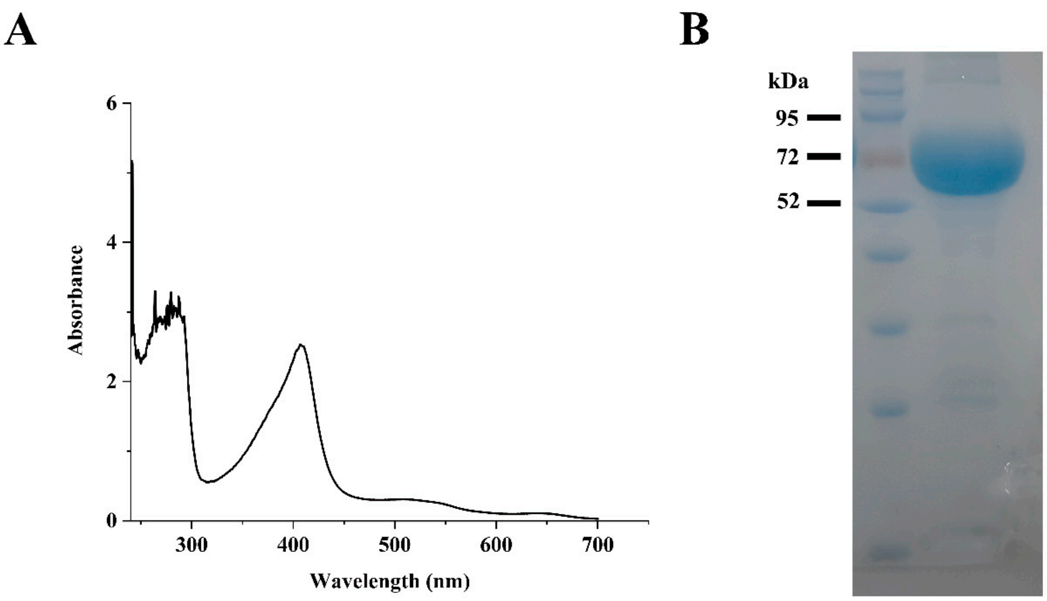

Figure S5. UV-Vis spectrum of CAT-3317 (A) and SDS-PAGE analysis of CAT-3317 (B).

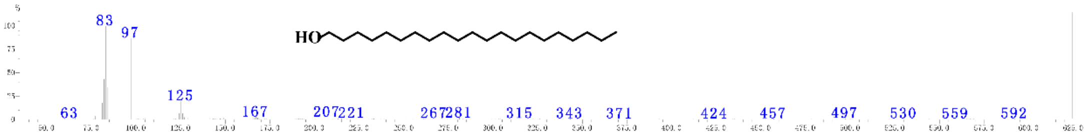

Figure S6. The mass spectrum and structural formula of the potential degradation product.
